# Fosmanogepix (APX001) Is Effective in the Treatment of Immunocompromised Mice Infected with Invasive Pulmonary Scedosporiosis or Disseminated Fusariosis

**DOI:** 10.1128/AAC.01735-19

**Published:** 2020-02-21

**Authors:** Sondus Alkhazraji, Teclegiorgis Gebremariam, Abdullah Alqarihi, Yiyou Gu, Zeinab Mamouei, Shakti Singh, Nathan P. Wiederhold, Karen J. Shaw, Ashraf S. Ibrahim

**Affiliations:** a Division of Infectious Diseases, The Lundquist Institute at Harbor-University of California Los Angeles (UCLA) Medical Center and St. John’s Cardiovascular Research Center, Torrance, California, USA; b University of Texas Health Science Center at San Antonio, San Antonio, Texas, USA; c Amplyx Pharmaceuticals, Inc., San Diego, California, USA; d David Geffen School of Medicine at UCLA, Los Angeles, California, USA

**Keywords:** APX001, APX001A, Gwt1, antifungal, *Fusarium*, *Scedosporium*, infection model, murine, manogepix, fosmanogepix, antifungal agents

## Abstract

There are limited treatment options for immunosuppressed patients with lethal invasive fungal infections due to *Fusarium* and *Scedosporium*. Manogepix (MGX; APX001A) is a novel antifungal that targets the conserved Gwt1 enzyme required for localization of glycosylphosphatidylinositol-anchored mannoproteins in fungi. We evaluated the *in vitro* activity of MGX and the efficacy of the prodrug fosmanogepix (APX001) in immunosuppressed murine models of hematogenously disseminated fusariosis and pulmonary scedosporiosis. The MGX minimum effective concentration (MEC) for *Scedosporium* isolates was 0.03 μg/ml and ranged from 0.015 to 0.03 μg/ml for *Fusarium* isolates. In the scedosporiosis model, treatment of mice with 78 mg/kg and 104 mg/kg of body weight fosmanogepix, along with 1-aminobenzotriazole (ABT) to enhance the serum half-life of MGX, significantly increased median survival time versus placebo from 7 days to 13 and 11 days, respectively. Furthermore, administration of 104 mg/kg fosmanogepix resulted in an ∼2-log_10_ reduction in lung, kidney, or brain conidial equivalents/gram tissue (CE). Similarly, in the fusariosis model, 78 mg/kg and 104 mg/kg fosmanogepix plus ABT enhanced median survival time from 7 days to 12 and 10 days, respectively. A 2- to 3-log_10_ reduction in kidney and brain CE was observed. In both models, reduction in tissue fungal burden was corroborated with histopathological data, with target organs showing reduced or no abscesses in fosmanogepix-treated mice. Survival and tissue clearance were comparable to a clinically relevant high dose of liposomal amphotericin B (10 to 15 mg/kg). Our data support the continued development of fosmanogepix as a first-in-class treatment for infections caused by these rare molds.

## Abstract (precis)

There are limited treatment options for immunosuppressed patients with lethal invasive fungal infections due to *Fusarium* and *Scedosporium*. Manogepix (MGX; APX001A) is a novel antifungal that targets the conserved Gwt1 enzyme required for localization of glycosylphosphatidylinositol-anchored mannoproteins in fungi. We evaluated the *in vitro* activity of MGX and the efficacy of the prodrug fosmanogepix (APX001) in immunosuppressed murine models of hematogenously disseminated fusariosis and pulmonary scedosporiosis.

## INTRODUCTION

Mycoses resulting from hyalohyphomycetes such as *Scedosporium* spp. and *Fusarium* spp. generally results in a poor prognosis, with the outcome related to the degree and persistence of immunosuppression (1, 2). Scedosporiosis comprises a wide range of clinical diseases, ranging from localized to disseminated infections in both immunocompromised and immunocompetent hosts (3). Although rare, these infections are often difficult to treat with currently available antifungal agents. Mortality can be as high as 80% in patients with invasive scedosporiosis, and Scedosporium apiospermum is the leading cause of eumycetoma in North America and Western countries (4). Lomentospora prolificans (formerly *Scedosporium prolificans*) is generally highly resistant to antifungal therapy, with mortality rates in immunocompromised patients similar to those reported for S. apiospermum (3). *Fusarium* spp. are major causes of superficial infections, including onychomycosis and keratitis, in immunocompetent individuals (5). Fusariosis can also cause serious hematogenously disseminated infections in severely immunocompromised patients, such as those with hematologic malignancies, which are often associated with poor outcomes (6). Due to the rarity of scedosporiosis and fusariosis, clinical trials for these diseases are problematic and the optimal antifungal therapy is unclear. In addition to surgical debridement of the infected tissue, the guidelines of the Infectious Diseases Society of America (IDSA) recommend voriconazole (VORI) or lipid-based amphotericin B (AMB) formulations as first-line therapy for patients with severe *Fusarium* infections, with posaconazole (POSA) as salvage therapy (7). Similarly, guidelines for *Scedosporium* include VORI or POSA as preferred agents for first-line therapy, but notably, some organisms are resistant to AMB (7). However, even with current standard-of-care treatments, patients with these diseases still have high mortality (80 to 100%), especially those with prolonged immunosuppression (3, 4, 8). More favorable clinical outcomes are generally associated with rapid diagnosis, surgical debridement of the infection, improvement in neutrophil counts, and early use of appropriate antifungal therapy, either alone or in combination. However, current antifungal treatments are often associated with safety and toxicity issues. Thus, alternative treatment options are needed.

Fosmanogepix (APX001) is a broad-spectrum first-in-class small-molecule antifungal that is currently in clinical development for the treatment of invasive fungal infections (9). Fosmanogepix is an N-phosphonooxymethyl prodrug that is rapidly and completely metabolized by host systemic phosphatases to the active moiety, manogepix (MGX; formerly APX001A) (10). MGX targets the highly conserved fungal enzyme Gwt1, which catalyzes an early step in glycosylphosphatidylinositol (GPI)-anchor biosynthesis (11). Interestingly, the related mammalian ortholog, PIGW, is not sensitive to MGX inhibition (12).

In a recent international SENTRY survey of 1,706 fungal clinical isolates from 2017, MGX demonstrated activity against a wide range of pathogenic yeast and molds (13). MIC_90_ values for yeasts were the following: *Candida* spp., 0.06 μg/ml; Cryptococcus neoformans, 0.5 μg/ml. The MEC_90_ value for *Aspergillus* spp. was 0.03 μg/ml. MEC values ranged from 0.015 to 0.06 μg/ml for 11 *Scedosporium* species isolates evaluated. Similarly, a previous report showed that the MGX MEC_90_ values for S. apiospermum and S. prolificans were 0.12 μg/ml, and the MEC_90_ values for all *Fusarium* spp. evaluated were 0.12 μg/ml (67 strains) (14). The spectrum of MGX is notable for activity against many less common but antifungal-resistant strains (13).

The efficacy of fosmanogepix has been demonstrated in multiple mouse models of invasive pulmonary and disseminated fungal infections, including those caused by *Candida*, *Coccidioides, Cryptococcus*, and *Aspergillus* spp. (15–18). In these studies, treatment with fosmanogepix resulted in increased survival and reduced colony counts of fungi in the lungs, kidney, and brain tissues of infected mice, as well as histological improvement (10, 15–17, 19). Several of these studies involved preadministration of 1-aminobenzotriazole (ABT), a nonselective suicide inhibitor of cytochrome P450 (CYP) enzymes (20), to increase the exposure and half-life of MGX in mice (17, 19). We have previously shown that 50 mg/kg ABT administered 2 h prior to fosmanogepix enhanced MGX exposures (area under the concentration-time curve [AUC]) 16- to 18-fold and enhanced serum half-life from ∼1 to 9 h, more closely mimicking human pharmacokinetic values (2 to 2.5 days) observed in phase 1 clinical studies in healthy volunteers (9, 16, 21).

In this study, we assessed the *in vitro* activity of MGX against agents of scedosporiosis, lomentosporiosis, and fusariosis. We evaluated the efficacy of fosmanogepix, at clinically relevant exposures, in two immunosuppressed murine models of (i) pulmonary scedosporiosis and (ii) hematogenously disseminated fusariosis. In these studies, we analyzed survival, tissue fungal burden as measured by a quantitative PCR (qPCR) assay, which assessed log_10_ conidial equivalents/gram tissue (CE), as well as histological improvement.

## RESULTS

### Antifungal susceptibility.

The activities of MGX and comparators were evaluated against a panel of 6 *Scedosporium*, 3 *Lomentospora prolificans*, and 10 *Fusarium* clinical isolates, including strains that were utilized in the efficacy models (Table 1). Susceptibility was evaluated using the CLSI M38-A2 broth microdilution method for filamentous fungi (22). The MGX MEC endpoint criteria were as described for the echinocandins. MIC values were determined for the comparators VORI, POSA, and AMB. The range of MGX concentrations evaluated in the antimicrobial susceptibility studies was 0.015 to 8.0 μg/ml. The MEC value of MGX was 0.03 μg/ml for all individual *Scedosporium* and *Lomentospora* isolates tested, including S. apiospermum DI16-478, used in the efficacy model. The MEC value of MGX ranged from ≤0.015 μg/ml to 0.03 μg/ml for the 10 F. solani isolates (Table 1). An additional 19 F. oxysporum strains were also evaluated, and all isolates demonstrated MGX MEC values of ≤0.015 μg/ml (data not shown). MIC_90_ values of comparator drugs for these strains were the following: 2 μg/ml AMB, 8 μg/ml VORI, and 8 μg/ml POSA (data not shown). These data are consistent with ranges of MGX MEC values reported previously for *Fusarium* spp. (0.015 to 0.5 μg/ml) and *Scedosporium* spp. (0.03 to 0.25 μg/ml) (14). The POSA and AMB MIC values against S. apiospermum DI16-478 were 0.5 μg/ml and 1.0 μg/ml, respectively, whereas the VORI and AMB MIC values against F. solani 95-2478 were 2 μg/ml (Table 1).

**TABLE 1:** Antifungal susceptibility profile of MGX and comparator agents

Clinical isolate	MIC/MEC (μg/ml)			
	MGX	POSA	VORI	AMB
Scedosporium apiospermum DI16-476	0.03	1.0	0.5	4.0
Scedosporium apiospermum DI16-477	0.03	0.5	ND^*b*^	2.0
Scedosporium apiospermum DI16-478^*a*^	0.03	0.5	1.0	1.0
Scedosporium boydii DI16-479	0.03	0.25	1.0	8.0
Scedosporium boydii DI16-480	0.03	2.0	1.0	4.0
Scedosporium boydii DI16-481	0.03	4.0	1.0	2.0
*Lomentospora prolificans* DI16-482	0.03	>16	>16	>16
*Lomentospora prolificans* DI16-483	0.03	>16	>16	8
*Lomentospora prolificans* DI16-484	0.03	>16	>16	>16
Fusarium solani 95-2478^*a*^	0.03	>16	2	2
Fusarium solani DI19-284	≤0.015	4	2	1
Fusarium solani DI19-285	≤0.015	8	2	0.25
Fusarium solani DI19-286	≤0.015	>16	16	1
Fusarium solani DI19-287	≤0.015	>16	>16	2
Fusarium solani DI19-288	≤0.015	>16	>16	0.5
Fusarium solani DI19-289	≤0.015	>16	8	1
Fusarium solani DI19-290	≤0.015	>16	>16	2
Fusarium solani DI19-291	≤0.015	>16	>16	0.5
Fusarium solani DI19-292	≤0.015	>16	16	2

a Strain used in the mouse infection model.

b ND, not determined.

### Fosmanogepix demonstrates efficacy in a highly immunocompromised pulmonary scedosporiosis model.

**FIG 1:**
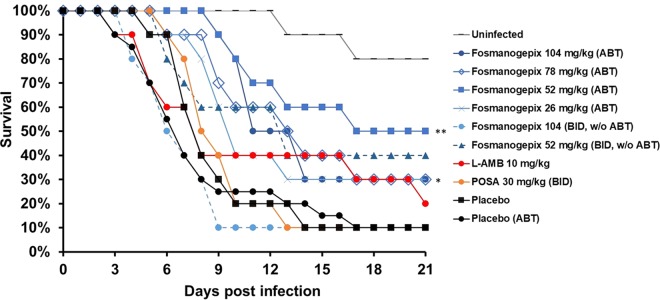
Survival of immunosuppressed mice infected with S. apiospermum DI16-478. ICR male mice (*n* = 10 to 20) were infected with an average inoculum of 3.0 × 10^7^ spores of S. apiospermum DI16-478 per mouse via inhalation. Treatment was initiated 16 h after infection and continued daily for 7 days. A dose of 50 mg/kg ABT was administered orally 2 h prior to fosmanogepix dose in all groups except the 52-mg/kg and 104-mg/kg (BID, without ABT) groups, as indicated. The nonparametric log-rank test was used to determine differences in survival times. *P* values versus placebo (ABT) were 0.004 (**) and <0.05 (*); *P* values versus placebo (without ABT) for POSA, L-AMB, and fosmanogepix (104 mg/kg and 52 mg/kg without ABT) did not achieve statistical significance (*P* > 0.16).

To assess the effect of fosmanogepix in the treatment of pulmonary scedosporiosis, ICR mice were immunosuppressed with cyclophosphamide (200 mg/kg) and cortisone acetate (500 mg/kg) on days −2 and +3 relative to infection. This treatment regimen results in ∼10 days of leukopenia with a total white blood cell count dropping from ∼13,0000/cm^3^ to almost no detectable leukocytes, as determined by the Unopette system (BD, NJ) (23). Mice were intratracheally infected with 3 × 10^7^ spores of S. apiospermum DI16-478 on day 0. This strain is susceptible to azoles as well as to micafungin and previously has been validated in a neutropenic mouse model of scedosporiosis (24). Treatment with placebo (diluent control), fosmanogepix (26, 52, 78, or 104 mg/kg, orally [p.o.]), POSA (30 mg/kg twice a day [BID], p.o.), or liposomal amphotericin B (L-AMB, 10 mg/kg, intravenous [i.v.]) began 16 h postinfection and continued daily for 7 or 11 days for fosmanogepix or POSA and 4 days for L-AMB. To extend the half-life of MGX, 4 of the mouse cohorts were also administered 50 mg/kg of the cytochrome P450 inhibitor 1-aminobenzotriazole (ABT) 2 h prior to fosmanogepix administration, as previously described (19) and as labeled in Fig. 1 and Table 2.

**TABLE 2:** S. apiospermum pulmonary infection median survival time

Treatment^*a*^	Median survival time (days)	*P* value (versus placebo)
Placebo	8	
Placebo (ABT)	7	0.566
Fosmanogepix		
26 mg/kg (ABT)	10	0.07
52 mg/kg (ABT)	17	0.004
52 mg/kg (w/o ABT)	13	0.16
78 mg/kg (ABT)	13	0.04
104 mg/kg (ABT)	11	0.03
104 mg/kg BID (w/o ABT)	6	0.36
POSA, 30 mg/kg BID	8	0.08
L-AMB, 10 mg/kg	8	0.65

a w/o, without.

**(i) Survival.** Survival of mice was assessed over 21 days (*n* = 10 to 20 mice/cohort). The median survival time for vehicle control cohorts with and without ABT were equivalent (*P* = 0.566): 7 and 8 days, respectively (Table 2). Neither POSA nor L-AMB prolonged median survival time versus the placebo (*P* > 0.5). In contrast, dosing regimens of 52, 78, and 104 mg/kg fosmanogepix plus ABT for 7 days significantly demonstrated prolonged median survival times of 17, 13, and 11 days, respectively (Table 2 and Fig. 1). Of note is that the surviving mice looked healthy. Further, these fosmanogepix treatments enhanced overall survival by day 21, when the experiment was terminated (30% to 50% for 52 to 104 mg/kg versus 10% for placebo or POSA). Survival after treatment with fosmanogepix for 7 days or 11 days was not different (data not shown). Dosing regimens yielding lower exposures (26 mg/kg with ABT, 52 mg/kg BID without ABT, and 104 mg/kg BID without ABT) did not result in statistically significant prolonged median survival times (*P* = 0.7, *P* = 0.16, and *P* = 0.36, respectively) (Table 2 and Fig. 1), consistent with a dose response.

**(ii) Tissue burden.** The effect of drug treatment on tissue fungal burdens was examined using qPCR to evaluate log_10_ conidial equivalents (CE)/gram of tissue. Mice (*n* = 10) were infected and treated as in the survival studies and then sacrificed on day +4 (8 h after the last treatment), and lungs (primary target organ), kidneys, and brains were processed and evaluated for CE/gram of tissue (25). In this experiment, we evaluated several doses above the highest dose used in the survival study (104 mg/kg plus ABT) in order to determine whether additional reductions in CE would be observed at higher doses. These higher doses (156, 208, and 264 mg/kg, all plus ABT) can only be used in studies in which mice are sacrificed at an early time point (e.g., 4 days) but not in longer-term survival studies due to mouse-specific toxicity observed in highly immunocompromised mice (18).

**FIG 2:**
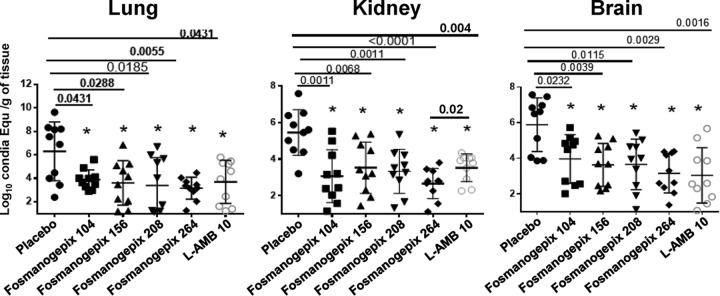
Reduction in tissue burden in the pulmonary scedosporiosis model. Tissue burdens in mice (10 per group) were measured at 4 days postinfection with S. apiospermum DI16-478. Male ICR mice were infected with 3.0 × 10^7^ spores via inhalation. Treatment was initiated 16 h postinfection and continued for 4 days. ABT (50 mg/kg) was administered orally 2 h prior to daily fosmanogepix dosing. Mice were sacrificed 8 h after the last dose, and lung, kidney, and brain were harvested and processed for tissue fungal burden by qPCR. *P* values are shown versus placebo control. None of the other intergroup comparisons reached statistical significance except for reduction in kidney burden for 264 mg/kg fosmanogepix (plus ABT) versus 10 mg/kg L-AMB (*P* = 0.02). Conidia Equ, conidia equivalents.

At all doses (104 to 264 mg/kg), fosmanogepix plus ABT significantly reduced tissue fungal burden in lung (*P* < 0.04) and brain (*P* < 0.007) compared to that in placebo-treated mice and were comparable to that of L-AMB treatment (*P* > 0.18) (Fig. 2). In kidney, all doses of 104 mg/kg to 264 mg/kg plus ABT significantly reduced fungal burden compared to placebo control (*P* < 0.007), but only the highest dose (264 mg/kg plus ABT) demonstrated statistical significance versus L-AMB treatment (*P* = 0.02) (Fig. 2). Thus, a dose-response curve generally was not observed at these higher dosing levels. All fosmanogepix treatments resulted in an ∼2-log reduction in lung, brain, and kidney CE.

**FIG 3:**
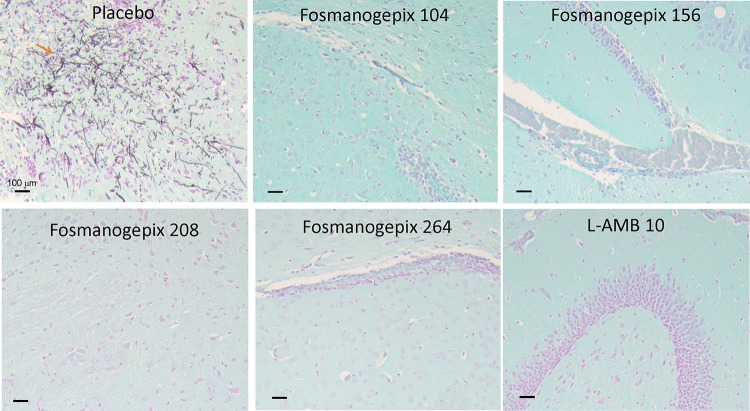
Histological examination of brain harvested from mice infected with S. apiospermum and treated with placebo, fosmanogepix, or L-AMB. Mice were infected and treated as described for Fig. 2. Harvested fixed brains were stained with GMS prior to microscopic examination. Notice the intact hyphae in the placebo control group. Treatment with fosmanogepix plus ABT or L-AMB resulted in normal brain architecture with no signs of fungal dissemination.

**(iii) Histological observations.** Brain tissues taken from placebo mice processed at the same time of the tissue fungal burden experiment (4 cohorts of 104, 156, 208, and 264 mg/kg plus ABT) and collected at day +4 postinfection (*n* = 3 mice/group) showed that S. apiospermum spores had disseminated from the lungs to the brain with fulminant growth and inflammation (Fig. 3) that coincided with neurological symptoms, including torticollis, spinning, and barrel rolling. In contrast, no signs of infection or inflammation could be detected in any of the brains collected from mice treated with fosmanogepix (104 to 264 mg/kg plus ABT) or L-AMB (10 mg/kg) (Fig. 3). These results confirm the similar efficacy of fosmanogepix and L-AMB in this scedosporiosis murine model. Surprisingly, we could not detect any signs of infection in the lung (the primary infection target), indicating the rapid dissemination of the infection to the brain and/or lack of fungal filamentation in this organ (data not shown).

### Fosmanogepix demonstrates efficacy in an immunocompromised disseminated fusariosis model.

We investigated the efficacy of fosmanogepix in the treatment of disseminated fusariosis, as previously described (26). Immunosuppression of ICR mice was performed as described above for the scedosporiosis model. Mice were infected with 8.1 × 10^2^ spores of F. solani 95-2478 (MEC, 0.03 μg/ml) by tail vein injection on day 0. This strain was from a patient blood sample and was previously used in neutropenic mouse models of disseminated fusariosis (26, 27). Treatment with placebo (diluent control), fosmanogepix (78 or 104 mg/kg, p.o.) plus 50 mg/kg ABT, L-AMB (15 mg/kg, i.v.), or VORI (40 mg/kg, p.o.) began 16 h postinfection and continued for 8 days for fosmanogepix or VORI and 4 days for L-AMB. To enhance the half-life of the molecules, 50 mg/kg ABT was administered 2 h prior to fosmanogepix, and grapefruit juice (50%) was added in the drinking water for VORI-treated mice (28). Mice were sacrificed on day +4 and organs processed to determine CE by qPCR.

**FIG 4:**
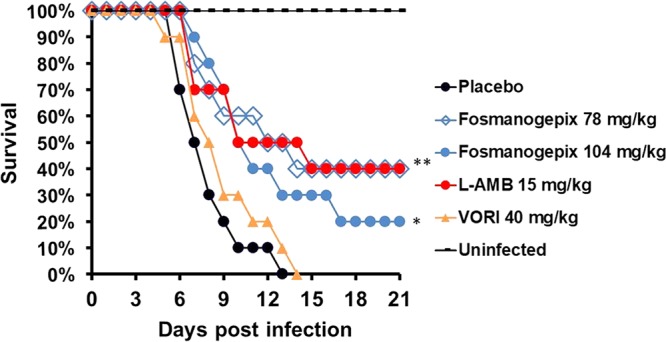
Survival of immunosuppressed mice infected with F. solani. ICR male mice (*n* = 10) were infected with an inoculum of 8.1 × 10^2^ spores per mouse via vein infection. Treatment was initiated 16 h after infection and continued daily for 8 days. Grapefruit juice (50%) was added into the drinking water of the VORI treatment group from day −2 to day +4 to enhance the drug half-life; 50 mg/kg ABT was administered daily 2 h prior to fosmanogepix dosing. *, *P* < 0.05; **, *P* < 0.002. *P* values were assessed versus the placebo control using the log rank test.

**(i) Survival.** Treatment of mice (*n* = 10/group) with fosmanogepix plus ABT or L-AMB significantly (*P* < 0.01) enhanced median survival time versus placebo (12 and 10 days for 78 and 104 mg/kg of fosmanogepix, 10 days for L-AMB treatment) (Table 3). Furthermore, fosmanogepix plus ABT and L-AMB treatments equally enhanced overall survival by day 21 when the experiment was terminated (40% for L-AMB or fosmanogepix at 78 mg/kg and 20% for fosmanogepix at 104 mg/kg) (Fig. 4). VORI was not effective in this model in terms of median survival time or overall survival by day 21 (Table 3 and Fig. 4).

**TABLE 3:** F. solani hematogenously disseminated infection model median survival times

Treatment	Median survival time (days)	*P* value (versus placebo)
Placebo	7	
Fosmanogepix, 78 mg/kg	12	0.002
Fosmanogepix, 104 mg/kg	10	0.01
VORI, 40 mg/kg	8	0.42
L-AMB, 15 mg/kg	10	0.001

**FIG 5:**
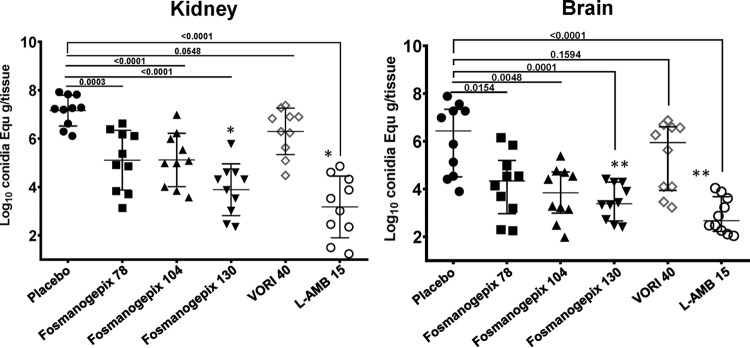
Reduction in kidney and brain tissue burden in a disseminated *Fusarium* model. Mouse kidney and brain tissue burdens in mice (*n* = 10) were measured at 4 days postinfection. Male ICR mice were infected and treated as described for Fig. 4 with a dose of 50 mg/kg ABT administered orally 2 h prior to daily fosmanogepix dosing. Mice were sacrificed 8 h after the last dose, and kidney and brain tissues were harvested and processed for fungal burden by qPCR. Fungal burden data (presented as medians ± interquartile ranges) were log_10_ transformed and evaluated using the nonparametric Wilcoxon rank sum test (Prism 5; GraphPad Software, Inc., San Diego, CA). *P* values are shown versus placebo control. (A and B) Intergroup comparisons. (A) For kidney, 130 mg/kg fosmanogepix was equivalent to L-AMB (*P* = 0.2853), and they both had *P* values of <0.05 (*) versus all other groups. (B) For brain, 130 mg/kg fosmanogepix, 104 mg/kg fosmaogepix, and L-AMB were equivalent (*P* > 0.06) and had *P* values of <0.05 (**) versus all other groups.

**(ii) Tissue burden.** Administration of L-AMB (15 mg/kg) and fosmanogepix (78 mg/kg, 104 mg/kg, and 130 mg/kg) plus ABT resulted in reductions in tissue burden CE that were statistically different from values for the placebo control (kidney, *P* < 0.0003; brain, *P* < 0.0154) (Fig. 5) (*n* = 10/group). Compared to placebo, treatment with 78, 104, or 130 mg/kg fosmanogepix plus ABT reduced kidney counts by 2.10, 2.21, and 3.14 log_10_ CE, respectively, while L-AMB treatment resulted in a 3.96-log_10_ reduction in kidney counts. L-AMB demonstrated activity that was equivalent to 130 mg/kg fosmanogepix in reduction of kidney CE (*P* = 0.3). Compared to placebo, treatment with 78, 104, or 130 mg/kg fosmanogepix plus ABT reduced brain counts by 2.10, 2.58, and 3.04 log_10_ CE, respectively, versus 3.76 log_10_ for L-AMB. L-AMB demonstrated activity that was statistically equivalent to both 104 mg/kg and 130 mg/kg fosmanogepix in reduction of brain CE (*P* ≥ 0.07). VORI did not demonstrate statistically significant reductions in CE versus placebo control in brain (0.48 log_10_ CE) or kidney (0.68 log_10_ CE), and fosmanogepix outperformed this azole in treating hematogenously disseminated murine fusariosis (Fig. 5).

**FIG 6:**
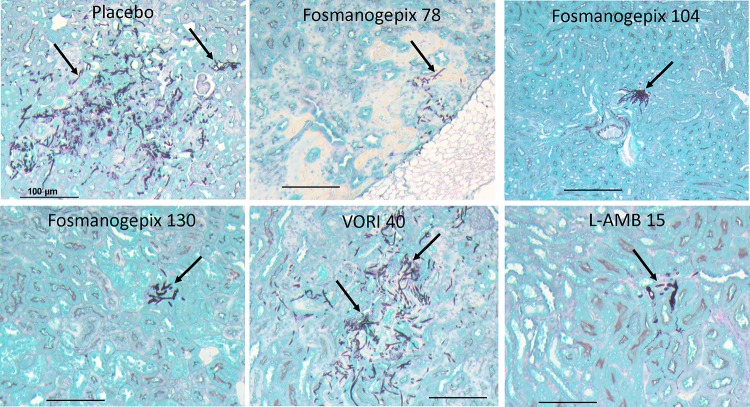
Histological examination of kidney tissue harvested from mice infected with F. solani and treated with placebo, fosmanogepix, VORI, or L-AMB. Mice were infected and treated as described for Fig. 5. Notice the diffused and fulminant filamentation in kidney tissue of F. solani in placebo- or VORI-treated mice. Fewer hyphae are evident in kidneys of mice treated with fosmanogepix or L-AMB.

**(iii) Histological observations.** Kidney tissues that were harvested from a parallel tissue burden experiment that utilized dosing regimens of 78, 104, and 130 mg/kg fosmanogepix (plus ABT) and 15 mg/kg L-AMB were also processed for histology (*n* = 3 mice/group). Consistent with the tissue fungal results, kidneys from placebo- or VORI-treated mice showed diffused and fulminant filamentation of F. solani (Fig. 6). In contrast, less diffusion of fungal hyphae was evident in kidneys treated with fosmanogepix at 78 mg/kg, while fungal elements were confined to a small abscess in kidneys treated with fosmanogepix at 104 mg/kg. Finally, kidneys harvested from mice treated with fosmanogepix at 130 mg/kg or L-AMB at 15 mg/kg had similar and consistently less infection than other groups (Fig. 6).

## DISCUSSION

There are limited treatment options for the management of hyalohyphomycosis, especially in the case of disseminated disease in highly immunocompromised patients. Fosmanogepix is an i.v. and orally available antifungal prodrug that is currently in clinical development for the treatment of life-threatening invasive fungal infections, including candidiasis and aspergillosis. The broad-spectrum nature of this first-in-class agent previously has been demonstrated *in vitro* as well as *in vivo* (15–18).

A previous study evaluated the efficacy of fosmanogepix in the treatment of disseminated fusariosis and pulmonary scedosporiosis using less immunocompromised models where 200 mg/kg of 5-fluorouracil was administered 5 or 6 days prior to infection (29). In the two survival experiments, the nadir of the neutrophil counts occurred approximately on the day of infection (30), with neutrophils recovering early during the course of the infection. The current regimen results in pancytopenia for at least 9 days from the first administered dose (23).

In this study, successful treatment outcomes utilized ABT plus oral dosing of 78 mg/kg or 104 mg/kg fosmanogepix, where total area under the plasma drug concentration-time curve (*t*AUC) values were ∼200 and ∼280 μg·h/ml (K. J. Shaw, unpublished), exposures that were shown in pharmacokinetic/pharmacodynamic (PK/PD) studies to be associated with A. fumigatus stasis and 1-log kill, respectively (31). To achieve ≥90% survival in the earlier study that utilized 5-fluorouracil with three-times-per-day (TID), intraperitoneal (i.p.) administration dosing regimens (without ABT) resulted in total *t*AUC values that ranged from ∼12 μg·h/ml (fusariosis) to ∼46 μg·h/ml (scedosporiosis), considerably lower than the exposures needed for efficacy in the current study, where animals were severely immunocompromised with a cyclophosphamide-cortisone acetate regimen. Consistent with the increased severity of the current model, fosmanogepix dosed at 104 mg/kg BID without ABT (*t*AUC of ∼50 μg·h/ml; Shaw, unpublished) did not result in statistically significant prolonged median survival times in the scedosporiosis model.

In terms of clinical relevance, the exposures reached in the current study (∼200 and ∼280 μg·h/ml) are consistent with exposures achieved in fosmanogepix phase 1 single- and multiple-ascending-dose studies (9, 21) and anticipated in future fosmanogepix clinical trials. Efficacy, as measured by median survival time versus placebo control, was observed in both the S. apiospermum pulmonary infection model and the F. solani disseminated model using both of these dosing regimens (*P* ≤ 0.05).

Although clinical guidelines for *Scedosporium* include VORI as the preferred agent for first-line therapy (7), the data from the pulmonary scedosporiosis model showed that there was no benefit in overall survival or in median survival time versus the placebo control (*P* = 0.08) of mice treated with a high dose of POSA (30 mg/kg BID), which resulted in exposures in mice that are approximately six times the clinically relevant dose (32). Of note is that this high dose of POSA was efficacious in a pulmonary aspergillosis model (18), highlighting the difficulty of successful treatment of pulmonary scedosporiosis despite the POSA MIC value against both molds being 0.5 μg/ml (Table 1) (18). Similarly, a high dose of 10 mg/kg L-AMB, which is typically used in invasive mold infection models as a comparator, was ineffective in extending survival in this model versus the placebo control (*P* = 0.65).

In the disseminated fusariosis model, the control antifungal agents utilized were also consistent with first-line standard of care for patients. In a previous study, Graybill et al. utilized grapefruit juice to enhance the half-life of VORI, resulting in the observation of prolonged survival and reduced numbers of CFU in a disseminated F. solani 95-2478 model when 10 mg/kg or 20 mg/kg VORI was administered (28). However, in this study, 40 mg/kg VORI plus grapefruit juice was not efficacious (*P* = 0.42). Although the same strain of F. solani and the same strain of mice (ICR) were used in the two studies, there are notable differences in the two experiments. The Graybill et al. study utilized a 3-log higher inoculum (8 × 10^5^ CFU/mouse versus 8.1 × 10^2^/mouse); however, the mice were immunocompromised with 150 mg/kg 5-fluorouracil on day −1 rather than utilizing the cyclophosphamide-cortisone acetate regimen utilized here. Consistent with the increased level of immunosuppression of the current model, the higher dose (40 mg/kg) of VORI was not effective in the current model. In contrast, in this study, mice administered a high dose of 15 mg/kg L-AMB demonstrated survival that was significantly different from that of the placebo control (*P* = 0.001) and was equivalent to fosmanogepix dosed at 78 and 104 mg/kg plus ABT (*P* > 0.5).

Histology and CFU number reduction were also evaluated in the S. apiospermum and F. solani models using dosing regimens that were of concentrations equal to or higher than those used in the survival study in order to determine whether greater organism clearance would be observed at higher doses. Compared to placebo control mice in the pulmonary scedosporiosis model, all doses of 104 mg/kg to 264 mg/kg fosmanogepix plus ABT resulted in a significant reduction in brain, lung, and kidney burdens. At these higher doses, no clear dose response was observed, since there was no significant difference between the fosmanogepix treatment regimens when evaluated pairwise, suggesting that maximal activity was achieved at the dose of 104 mg/kg fosmanogepix plus ABT or was outside the dynamic range of the assay.

Consistent with the survival results using the disseminated fusariosis model, compared to the placebo control group, significant reductions in brain and kidney fungal burden were observed for fosmanogepix (78 mg/kg, 104 mg/kg, and 130 mg/kg) plus ABT and 15 mg/kg L-AMB but not for VORI. In brain, again no statistical significance was observed between fosmanogepix dosing groups; however, kidney burden was statistically lower for the highest fosmanogepix dosing group (130 mg/kg) and equivalent to that for L-AMB. The activity of fosmanogepix was also confirmed by the lack of detection of any fungal elements in the brains of mice with scedosporiosis and with the reduced fungal elements seen in kidneys of mice infected with F. solani.

In this study, we showed that the active moiety MGX was highly active against S. apiospermum, S. boydii, *L. prolificans*, and F. solani *in vitro*. Using highly immunocompromised mouse models of pulmonary scedosporiosis and disseminated fusariosis, we demonstrated that the efficacy of fosmanogepix was equivalent to or better than the current standard of care of antifungal agents. Findings from both oral and i.v. fosmanogepix phase 1 clinical studies have shown favorable PK, allowing once-daily dosing with high bioavailability (∼90%) and no food effect (9, 21). Given the previous demonstration of efficacy across a broad range of yeasts and mold mouse models, including *Candida* (15, 16, 19) and *Aspergillus* (18, 31), activity against azole- and echinocandin-resistant isolates of *Aspergillus* species (*Cyp51* and *Fks1*, respectively) (31), as well as the data described here for *Fusarium* and *Scedosporium*, further investigations into the development of this first-in-class agent for invasive fungal infections is highly warranted, especially against difficult-to-treat infections.

## MATERIALS AND METHODS

### Microorganisms.

Nine clinical isolates of *Scedosporium/Lomentospora* and 9 clinical isolates of Fusarium solani were obtained from the Fungus Testing Laboratory at the University of Texas Health Sciences Center at San Antonio (UTHSCSA) (Table 1). Fusarium solani 95-2478 is a blood isolate provided by P. Ferrieri (University of Minnesota) (26). Fungal strains used in this study were routinely grown on Sabouraud dextrose agar plates for 5 to 10 days until confluent at 37°C. Conidia were collected by flooding the plates with sterile phosphate-buffered saline containing 0.01% (vol/vol) Tween 80. The conidia were concentrated by centrifugation and washed in the same buffer, diluted, and counted using a hemocytometer.

### Antifungal agents.

For *in vitro* studies, the active moiety MGX (Amplyx Pharmaceuticals) was used along with AMB (Fisher Scientific, Hampton, NH), POSA (Sigma-Aldrich Corp., St. Louis, MO, USA), and VORI (Sigma-Aldrich Corp., St. Louis, MO, USA). For efficacy studies, the water-soluble N-phosphonooxymethyl prodrug fosmanogepix (Amplyx Pharmaceuticals) was used. The final prodrug solution was in 5% dextrose and dosed orally (p.o.) per gram of mouse body weight. A 5-mg/ml solution of ABT (Fisher Scientific, Hampton, NH) in water was administered orally 2 h prior to infection as 10 μl per gram of mouse body weight, resulting in a dose of 50 mg/kg, and this served as the placebo plus ABT group. For *in vivo* efficacy studies, POSA (Merck & Co., Inc., Rahway, NJ) was purchased as an oral suspension (200 mg/5 ml) and kept at room temperature. L-AMB (Gilead Science, Foster City, CA) and VORI (50-mg tablet from Ajanta Pharma USA, Bridgewater, NJ) were obtained from local pharmacies. The final L-AMB solution was dissolved in 5% dextrose and administered intravenously, while crushed VORI tablets were suspended in irrigation water and administered to mice by oral gavage.

### *In vitro* testing.

The *in vitro* susceptibility of manogepix (drug concentrations of 0.015 to 8.0 μg/ml) against agents of scedosporiosis and fusariosis was evaluated using the Clinical Laboratory and Standards Institute (CLSI) M38-A2 method using minimum effective concentration (MEC) endpoints for the echinocandins (22). Three clinical isolates each of Scedosporium apiospermum, S. boydii, and *Lomentospora prolificans* were evaluated along with 10 strains of F. solani. MIC values were determined for POSA, VORI, and AMB (22). All *in vitro* testing was conducted with drug powders with known potencies.

### Efficacy models.

ICR mice (Envigo) were immunosuppressed with cyclophosphamide (200 mg/kg) and cortisone acetate (500 mg/kg) on days −2 and +3 relative to infection (*n* = 10/group). To prevent bacterial infection, enrofloxacin (50 μg/ml) was added to the drinking water from day −3 to day 0. Ceftazidine (5 μg/dose/0.2 ml) replaced Baytril treatment on day 0 and was administered daily by subcutaneous injection from day 0 until day +8. All drug treatments were initiated 16 h postinfection and continued for 8 consecutive days, given by oral gavage for POSA, VORI, or fosmanogepix, but L-AMB was given intravenously through day +4. To extend the half-life of MGX, mice were administered 50 mg/kg the cytochrome P450 inhibitor ABT 2 h prior to fosmanogepix administration for most of the cohorts. The prodrug fosmanogepix was dosed at 26 mg/kg (with ABT), 52 mg/kg (with and without ABT), 78 mg/kg (with ABT), 104 mg/kg (with and without ABT), 156 mg/kg (with ABT), 208 mg/kg (with ABT), and 264 mg/kg (with ABT) in the various experiments. Using a conversion factor of 1.3 to account for the methyl phosphate group in the prodrug, the doses were equivalent to MGX at 20 mg/kg, 40 mg/kg, 60 mg/kg, 80 mg/kg, 120 mg/kg, 160 mg/kg, and 203 mg/kg, respectively.

### (i) Pulmonary S. apiospermum.

The immunosuppressed mice were challenged with 3.0 × 10^7^ spores of S. apiospermum DI16-478 through intratracheal instillation of 25 μl after sedation with isoflurane gas (33). Immediately after infection, a subset of mice was sacrificed and lungs were removed to determine infection inoculum by quantitative culturing (*n* = 10/group). For S. apiospermum survival studies, treatment with placebo (diluent control), fosmanogepix (26, 52, 78, or 104 mg/kg, p.o.), POSA (30 mg/kg, BID or p.o.; representing ∼6 times the human equivalent dose [32]), or L-AMB (10 mg/kg, i.v.) begun 16 h postinfection and continued for 7 days for fosmanogepix or POSA and 4 days for L-AMB. Mice were monitored for 21 days. To assess tissue fungal burden, mice were sacrificed on day +4 and organs processed for conidial equivalents (CE) by qPCR using actin primers (forward [F], CCCTTGACTTTGAGCAGGAG; reverse [R], CTCAAGACCGAGGACAGAGG). Mouse DNA was detected with the glyceraldehyde-3-phosphate dehydrogenase (GAPDH) primers (F, AGGCAACTAGGATGGTGTGG; R, TTGATTTTGGAGGGATCTCG).

### (ii) Disseminated F. solani.

Mice were infected with a targeted inoculum of 8.1 × 10^2^ cells of F. solani by tail vein injection. For survival studies, treatment with placebo (diluent control), fosmanogepix (78 or 104 mg/kg, p.o.) plus ABT, L-AMB (15 mg/kg, i.v.), or VORI (40 mg/kg, p.o.) began 16 h postinfection and continued for 8 days for fosmanogepix or VORI and 4 days for L-AMB (*n* = 10/group). To enhance the half-life of VORI, grapefruit juice (Ocean Spray) was added to the drinking water to a final concentration of 50% and was given to VORI-treated mice. To assess tissue fungal burden, mice were infected and treated as described above, except that mice were sacrificed on day +4 (4 days of treatment), 8 h following the last treatment (*n* = 10/group). Organs were processed for CE by qPCR using 28S primers (F, TAAATGGACCAGGGCGCAAA; R, AGAGGGAACGAGATGGGTT).

For both models, tissues harvested from mice to assess fungal burden were also processed for histopathological examination. Briefly, tissues were fixed in 10% zinc-buffered formalin, paraffin embedded, sectioned, and stained with Grocott’s methenamine silver (GMS) stain for microscopic examination (*n* = 3/group).

### Statistical analysis.

The nonparametric log rank test was used to determine differences in survival times. Differences in lung, kidney, and brain CFU numbers were compared by the nonparametric Wilcoxon rank sum test. A *P* value of <0.05 was considered significant.

All animal-related study procedures were compliant with the Animal Welfare Act, the *Guide for the Care and Use of Laboratory Animals* (34), and the Office of Laboratory Animal Welfare and were conducted under an IACUC-approved protocol by The Lundquist Institute at Harbor-UCLA Medical Center.
